# Supplementary figures and images for: Transcriptomic and Phenotypic Analysis of CRISPR/Cas9-Mediated gluk2 Knockout in Zebrafish
Source: Genes (Basel). 2022 Aug 13;13(8):1441. doi: 10.3390/genes13081441 (PMC9408333; doi:10.3390/genes13081441)

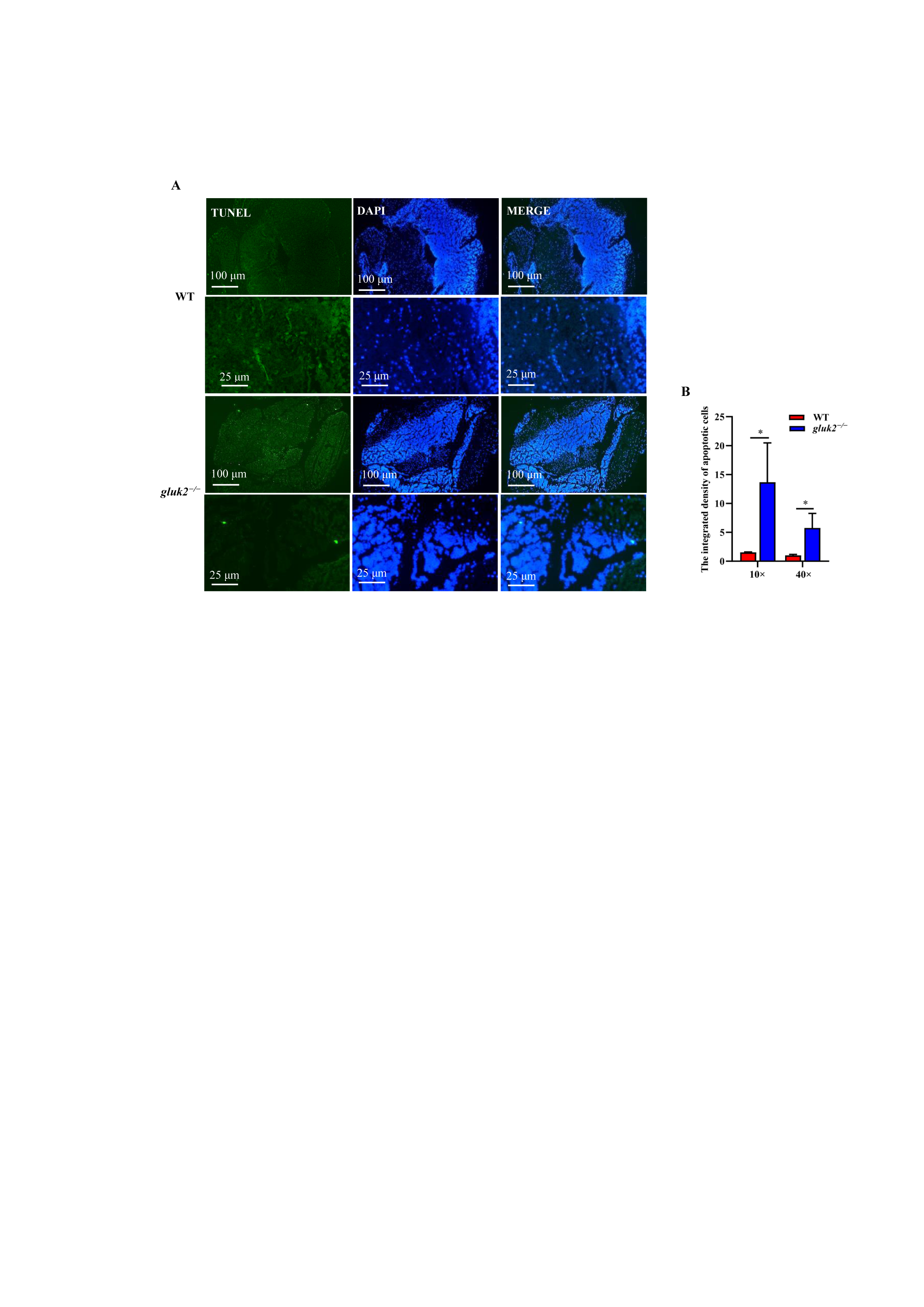

Supplement: Supplementary file 1 [file genes-13-01441-s001.zip › genes-1809357-supplementary.tif]
